# Mobile internet use, physical exercise and depression levels: mediating mechanism and empirical evidence

**DOI:** 10.3389/fpubh.2026.1830540

**Published:** 2026-06-18

**Authors:** Yue Sun, Zhenghao Hou

**Affiliations:** 1 Department of Sports Management, Yeungnam University, Gyeongsan-si, Republic of Korea; 2 Beijing Institute of Technology, Beijing, China

**Keywords:** depression, empirical evidence, exploratory mediation analysis, mobile internet use, physical exercise

## Abstract

**Introduction:**

Mental health has increasingly become a central component of national health agendas worldwide, aligning with the World Health Organization’s commitment to comprehensive and equitable health for all. This study examines the effect of mobile internet use on residents’ depressive symptoms and explores the behavioral mechanisms underlying this relationship.

**Methods:**

Using nationally representative panel data from the China Family Panel Studies (CFPS) for 2020–2022, this study employs a two-way fixed-effects model to empirically examine the effect of mobile internet use on residents’ depressive symptoms. Additional analyses are conducted to address potential endogeneity, explore the mediating role of physical exercise frequency, and examine heterogeneity across population subgroups.

**Results:**

The results show that mobile internet use significantly reduces depressive symptoms, and this finding remains robust after accounting for potential endogeneity. Further exploratory mediation analysis suggests that physical exercise frequency may serve as an important behavioral pathway through which mobile internet use alleviates depressive symptoms. Heterogeneity analyses further reveal that the depression-reducing effect of mobile internet use is more pronounced among vulnerable groups, including individuals with abnormal BMI, non-agricultural workers, workers in non-knowledge-intensive industries, and those without pension security.

**Discussion:**

Overall, these findings have important policy implications for promoting equitable digital access, designing targeted mental health interventions for vulnerable populations, and institutionalizing internet-enabled health promotion strategies.

## 1 Introduction

Depression has emerged as a critical global public health challenge and is consistently identified as a leading contributor to functional disability and health loss across populations. According to the World Health Organization, depressive disorders contribute substantially to the global burden of disease and are associated with persistent functional impairment, reduced quality of life, and high socioeconomic costs (1–3). Over recent decades, the prevalence of depression has continued to rise, with onset occurring at younger ages and the condition affecting a broader segment of the population, thereby placing increasing pressure on public health systems worldwide (4, 5).

In China, rapid socioeconomic transformation, a faster pace of life, and intensified competition have increased psychological pressure across age groups (6). Depression in the Chinese population is often chronic and under-recognized, which hinders timely detection and intervention and further exacerbates its adverse effects on individual well-being, work productivity, and social functioning (7, 8). Recognizing the severity of this challenge, national policy initiatives such as the *Healthy China 2030 Planning Outline* emphasize the importance of strengthening mental health services and promoting effective strategies for psychological health enhancement (9).

In this context, the rapid diffusion of digital technologies, particularly mobile internet technologies, has profoundly reshaped individuals’ daily lives, social interactions, and health-related behaviors (10–13). Mobile internet use has become deeply embedded in everyday activities, enabling unprecedented opportunities to access information, social networks, and digital services. Emerging evidence suggests that digital technologies may play an increasingly important role in shaping psychological well-being by facilitating social connection, reducing information barriers, and supporting health management practices (14–16). At the same time, mobile internet platforms provide new channels for health-related information dissemination, behavioral guidance, and emotional support, potentially offering novel tools for mental health promotion (17, 18).

However, the mental health implications of mobile internet use remain complex and contested. While some studies indicate that internet use can alleviate depressive symptoms by enhancing social connectedness and access to health resources (19–21), others raise concerns about information overload, excessive dependence, and digital inequality, which may undermine psychological well-being under certain conditions (22–24). These mixed findings suggest that the relationship between mobile internet use and depressive symptoms is unlikely to be purely direct and may be shaped by intermediate behavioral and contextual mechanisms.

Among potential behavioral pathways, physical exercise has been widely recognized as an effective non-pharmacological intervention for alleviating depressive symptoms (25, 26). A substantial body of evidence demonstrates that regular physical activity contributes to improved mental health through both physiological mechanisms, such as the release of endorphins and dopamine, and psychological processes, including emotional regulation, distraction from negative thoughts, and enhanced self-efficacy (27–30). Nevertheless, participation in physical exercise is uneven across populations, often constrained by informational, motivational, and environmental barriers (31–34).

In this context, mobile internet use may serve as an important facilitator of physical exercise by lowering access costs, providing exercise-related information and digital fitness tools, and fostering online social interactions that encourage sustained behavioral engagement (35, 36). As mobile internet technologies continue to integrate into daily life, understanding whether and how they influence depression through behavioral pathways, such as physical exercise, becomes increasingly important.

Taken together, the existing evidence highlights the growing relevance of mobile internet use to mental health and underscores the need for a more nuanced examination of its underlying mechanisms. Rather than focusing solely on the direct relationship between internet use and depression, it is essential to investigate the behavioral transmission processes through which digital technologies may shape psychological outcomes. Addressing this gap can provide a more comprehensive understanding of how mobile internet use contributes to mental health promotion in an increasingly digitalized society.

## 2 Literature review

### 2.1 Mobile internet use and depression

With the rapid expansion of digital technologies, a growing body of literature has examined the relationship between internet use and mental health, particularly depressive symptoms. Early studies emphasized the potential psychological risks associated with excessive or problematic internet use, suggesting that overuse may increase social isolation, disrupt daily routines, and exacerbate depressive tendencies (22, 23). More recent meta-analytic evidence further indicated that problematic internet use is significantly associated with adverse mental health outcomes, especially among vulnerable populations (24).

However, a growing number of studies adopt a more nuanced perspective, arguing that internet use is not inherently harmful and may, under certain conditions, have protective effects on mental health. Evidence from longitudinal and observational studies suggests that internet use may reduce depressive symptoms through multiple behavioral pathways, including improved access to information, broader interpersonal social networks, and greater opportunities for emotionally supportive interactions (15, 19, 20, 37). In particular, mobile internet use—characterized by portability, immediacy, and high-frequency engagement—has been found to play a distinctive role in shaping psychological well-being by integrating digital interactions into everyday life (14, 38).

Recent research further highlights that mobile internet technologies provide not only entertainment and information but also access to health-related services and psychological resources. Through social media platforms, online communities, and digital health tools, individuals can obtain emotional support, coping strategies, and mental health information at relatively low cost (16, 21). These functions suggest that mobile internet use may serve as a psychological resource rather than a burden, particularly in contexts of heightened social stress and limited offline support.

Despite these advances, existing findings remain mixed, reflecting substantial heterogeneity across populations, usage patterns, and social contexts. This inconsistency implies that the effect of mobile internet use on depression is unlikely to be purely direct and may operate through intermediate behavioral mechanisms that have not yet been sufficiently explored.

### 2.2 Physical exercise and depression

Physical exercise has long been recognized as an effective and low-cost strategy for alleviating depressive symptoms. Considerable empirical evidence demonstrates that regular physical activity contributes to improved mental health through multiple physiological pathways, notably including the stimulation of endorphin and dopamine release, the regulation of stress hormones, and the enhancement of neural plasticity (27, 30, 39). Meta-analyses and systematic reviews consistently report that both acute and long-term exercise interventions are associated with significant reductions in depressive symptoms across different populations (28, 29, 40).

Beyond the domain of biological mechanisms, psychological explanations emphasize the role of exercise in emotional regulation and cognitive restructuring. Physical activity can divert attention from negative thoughts, foster a sense of accomplishment, and enhance self-efficacy, all of which are crucial for alleviating depressive symptoms (29, 41, 42). From the perspective of Behavioral Activation Theory, depression is often characterized by reduced engagement in rewarding activities, while increased participation in positive behaviors, such as exercise, can disrupt this vicious cycle and improve emotional states (43).

However, despite its well-documented mental health benefits, participation in physical exercise remains unevenly distributed. Individual differences in motivation, access to information, time constraints, and environmental conditions often limit regular engagement in physical activity. These barriers highlight the importance of external facilitators that can lower participation costs and support sustained exercise behavior.

### 2.3 Mobile internet use and physical exercise

Recent studies suggest that mobile internet use may facilitate physical exercise by reshaping individuals’ behavioral environments. Mobile internet technologies provide convenient access to exercise-related information, fitness applications, online coaching, and digital health platforms, thereby reducing informational and organisational barriers to physical activity (35, 36). Through real-time feedback, activity tracking, and personalized recommendations, digital tools can enhance individuals’ awareness of their health behaviors and promote behavioral self-regulation (44).

Moreover, social features embedded in mobile internet platforms, such as online exercise communities, social media challenges, and peer interaction, can strengthen motivation and sustain long-term engagement in physical activity (45, 46). From the perspective of “Self-Determination Theory,” such digital environments support individuals’ basic psychological needs for autonomy, competence, and relatedness, thereby fostering intrinsic motivation for exercise participation (47). Empirical evidence indicates that internet-based fitness tools and social media interventions are positively associated with higher levels of physical activity and improved exercise adherence (48, 49).

Taken together, these findings suggest that mobile internet use indirectly influences mental health outcomes by promoting health-enhancing behaviors, particularly physical exercise. However, existing studies have largely examined these relationships in isolation, with limited attention to the integrated behavioral pathways linking mobile internet use, exercise behavior, and depression. Moreover, the effect of mobile internet use on physical exercise may depend on the specific mode of use. For instance, active, passive, and compulsory internet use may generate different behavioral incentives and may therefore have heterogeneous implications for health behaviors and depressive symptoms. Accordingly, the heterogeneity analysis uses employment in knowledge-intensive occupations as a proxy for high-frequency, active, and collaborative internet use, thereby indirectly examining whether patterns of internet use moderate the effect of mobile internet use on depressive symptoms.

### 2.4 Research gap

Although prior research has established separate links among mobile internet use, physical exercise, and depression, systematic investigation of the interrelationships among these variables remains limited.

First, most existing studies rely on cross-sectional designs, which limit causal inference and fail to establish the temporal ordering among the variables of interest (20, 38, 50–52). Although some studies have adopted longitudinal or panel data to strengthen causal interpretation, endogeneity concerns arising from reverse causality and omitted variable bias remain insufficiently addressed (19, 53).

Second, existing studies have primarily explained the relationship between mobile internet use and depressive symptoms from the perspective of social resources, including social support, interpersonal relationships, social networks, volunteer participation, and broader forms of social participation (37, 50–53). In contrast, relatively limited attention has been paid to health-promoting behaviors, particularly physical exercise, as an indirect pathway linking mobile internet use to mental health outcomes. Although Chen et al. (38) found that self-rated physical exercise partially mediated the relationship between digital lifestyles and mental health, their reliance on cross-sectional data and the absence of causal identification strategies limit the robustness of this finding and leave the directionality of the relationship uncertain. Similarly, Teng (17) employed an instrumental variable approach using CFPS 2020 data to address endogeneity concerns, but the study focused primarily on trust and happiness as mediating pathways rather than physical exercise as a behavioral mechanism.

Third, the protective effects of mobile internet use against depression across socially and economically vulnerable groups remain underexplored. Differences in physical health conditions, employment environments, and social security status may influence both access to digital technologies and the psychological benefits derived from them. However, most existing studies treat internet users as a relatively homogeneous population and pay limited attention to structural inequalities across subgroups (17, 52).

Our study addresses these knowledge gaps. First, it employs nationally representative panel data from 2020 to 2022 and applies a two-way fixed-effects model to control for time-invariant individual heterogeneity, thereby providing a stronger basis for causal inference than cross-sectional approaches. Second, the study adopts an instrumental variable strategy by using the internet access rate of other community residents as an instrument to mitigate potential endogeneity concerns. Third, it explicitly incorporates physical exercise as a mediating behavioral pathway linking mobile internet use to depressive symptoms and further investigates heterogeneous effects across vulnerable subpopulations defined by BMI status, occupational type, and pension coverage.

## 3 Conceptual framework and hypotheses

**Figure 1:**
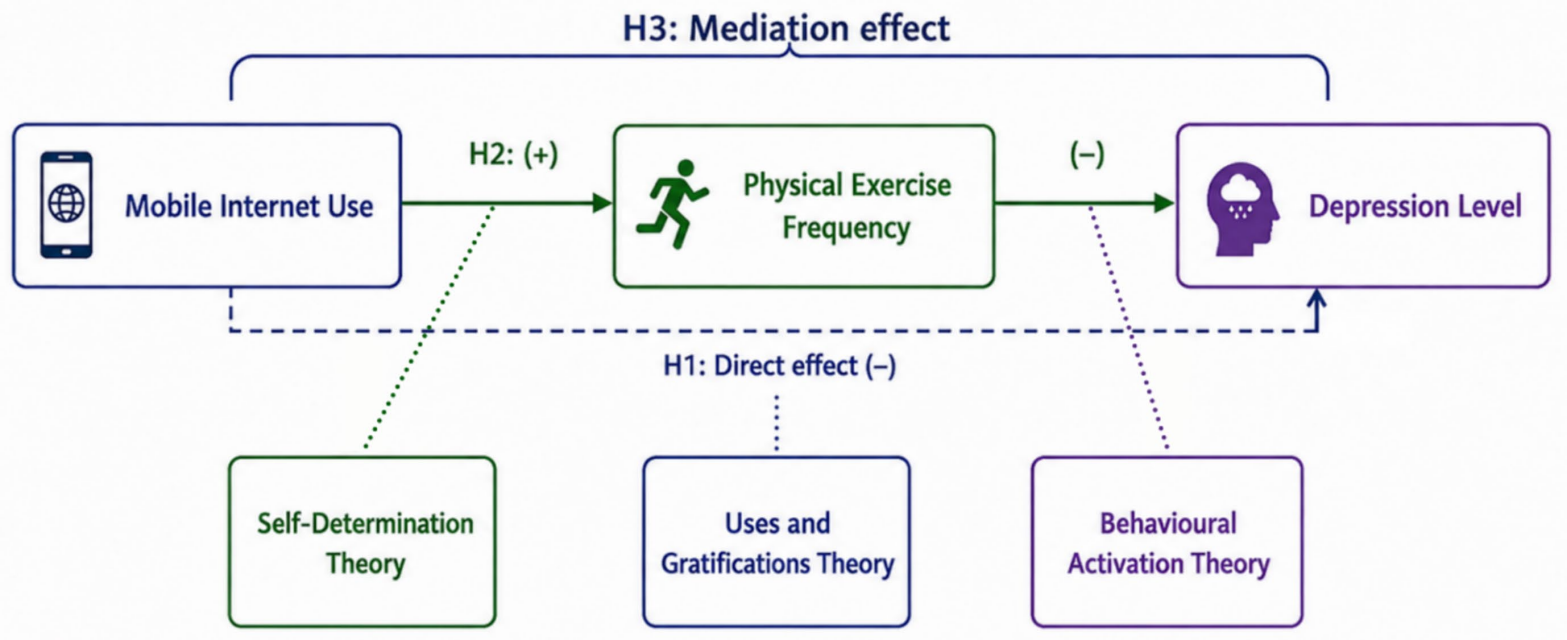
Conceptual framework and hypotheses.

This study develops an integrated conceptual framework grounded in three complementary theoretical perspectives, Uses and Gratifications Theory, Self-Determination Theory, and Behavioral Activation Theory, to explain the mechanisms through which mobile internet use influences depressive symptoms. Uses and Gratifications Theory explains the direct pathway through which mobile internet use reduces depression by illustrating how individuals actively use digital resources to satisfy their informational and social needs; Self-Determination Theory clarifies the motivational processes through which mobile internet use promotes and sustains participation in physical exercise; and Behavioral Activation Theory explains how increased exercise disrupts the behavioral inhibition–negative affect cycle underlying depression. Taken together, these three theories support a model in which mobile internet use influences depressive symptoms both directly and indirectly through the mediating role of physical exercise frequency. The conceptual framework is illustrated in Figure 1.

### 3.1 Mobile internet use directly reduces depression

Uses and Gratifications Theory posits that individuals are active media users who deliberately engage with digital technologies to satisfy specific psychological and social needs, including the acquisition of health-related information, emotional support, and social connection (54). By enabling individuals to access online platforms, social networks, and digital services anytime and anywhere, mobile internet use substantially reduces the cost of meeting these needs and strengthens perceived social connectedness (55, 56). Such improvements in access to information and social support may directly alleviate psychological stress, loneliness, and uncertainty, key precursors of depressive symptoms, thereby positioning mobile internet use as a psychological resource that helps reduce the risk of depression (15, 19, 20). Accordingly, the following hypothesis is proposed:

*H1*: Mobile internet use is negatively associated with individuals’ depressive symptom levels.

### 3.2 Mobile internet use promotes physical exercise participation

Self-Determination Theory posits that sustained engagement in any health behavior requires the fulfilment of three basic psychological needs: autonomy, competence, and relatedness (57). Mobile internet technologies are particularly well suited to satisfying these needs in the context of exercise behavior. Digital fitness applications, online coaching platforms, and exercise-tracking tools reduce informational and organisational barriers to physical activity, thereby enhancing individuals’ sense of competence and supporting autonomous motivation (35, 36). At the same time, social features embedded in mobile platforms, such as online exercise communities, peer challenges, and real-time social interaction, strengthen individuals’ sense of relatedness and help sustain long-term behavioral engagement (45, 46). Through these mechanisms, mobile internet use helps create the motivational conditions necessary to initiate and maintain regular physical exercise. Accordingly, the following hypothesis is proposed:

*H2*: Mobile internet use is significantly and positively associated with the frequency of physical exercise.

### 3.3 Physical exercise mediates the effect of mobile internet use on depression

Behavioral Activation Theory conceptualises depression as a self-reinforcing cycle characterised by reduced engagement in rewarding activities, the accumulation of negative affect, and progressive social withdrawal (43). As a positive health behavior, physical exercise can disrupt this cycle through neurochemical regulation, including the release of endorphins and dopamine, as well as through emotional distraction and enhanced self-efficacy (27–29, 39). By promoting exercise participation through the motivational pathways identified above, mobile internet use may indirectly alleviate depression, with physical exercise serving as the behavioral pathway linking digital engagement to psychological outcomes. It should be noted that, given the observational nature of the data, this mediation hypothesis is exploratory and is intended to identify a plausible behavioral pathway rather than establish strict causal mediation. Accordingly, the following hypothesis is proposed:

*H3*: Physical exercise frequency serves as a potential mediating pathway linking mobile internet use to depression levels.

## 4 Method

### 4.1 Data

This study uses microdata from the China Family Panel Studies (CFPS), conducted by the Institute of Social Science Survey (ISSS) at Peking University, together with the corresponding indicator system. The CFPS covers all provinces in China except Hong Kong, Macao, and Taiwan, and adopts a multistage, stratified, probability-proportional-to-size sampling design, ensuring strong national representativeness and reliability. In the sample processing stage, respondents aged 18–70 were selected as the analysis population. Samples with missing or abnormal values in key variables—such as depression scores, physical exercise frequency, and mobile internet use—were excluded, and extreme values in the calculated Body Mass Index (BMI) variable were removed to ensure the robustness of the estimates. To balance data representativeness with availability, this study uses CFPS data from 2020 to 2022 to construct an unbalanced panel dataset, yielding a total of 16,901 valid observations.

The dependent variable is depressive symptoms, measured using two indicators: cesd20sc (the 20-item Center for Epidemiologic Studies Depression Scale score) and cesd8 (the 8-item Center for Epidemiologic Studies Depression Scale score). Depression is one of the core manifestations of impaired mental health and one of the most widely used indicators for assessing mental health in empirical research. The two measures employed in this study are both derived from the Center for Epidemiologic Studies Depression Scale (CES-D), which is designed to evaluate individuals’ depressive symptoms. As a widely used and well-validated instrument in mental health research, the CES-D is consistent with established practices in the literature. Both measures capture the severity of depressive symptoms through numerical scores, with higher scores indicating more severe depressive symptoms.

The explanatory variables in this study are “use of mobile devices to access the internet” and “internet use.” The primary explanatory variable, “use of mobile devices to access the internet,” focuses on online activity conducted through mobile terminals such as smartphones and tablets. A value of 1 indicates that the respondent accesses the internet via such devices, while 0 indicates no such behavior. The “internet use” variable captures internet activity within a broader scope, covering all types of terminals—including mobile devices and computers—with 1 representing use and 0 representing non-use. Together, these indicators capture respondents’ patterns of internet access.

The mediating variable in this study is “frequency of physical exercise in the past 12 months,” which is divided into eight levels. Corresponding to the eight response options in the questionnaire (“How often did you participate in physical exercise or fitness activities in the past 12 months?”—never; less than once per month on average; more than once per month but less than once per week; 1–2 times per week on average; 3–4 times per week on average; 5 or more times per week; once per day; twice or more per day), values from 0 to 7 are assigned sequentially. Higher values represent higher exercise frequency.

Depressive symptoms are influenced by factors such as age, physical condition, employment, family circumstances, and economic status. Therefore, this study controls for a range of variables, including gender, age, years of education, marital status, household registration type, employment status, household size, and health status, the latter measured by self-rated health and whether BMI falls within the normal range. In addition, to control for fixed regional differences that may affect the results, this study also incorporates county-level regional attributes and year variables.^1^ The specific definitions of all variables are presented in Table 1.

**Table 1:** Variable definitions.

Variable type	Variable meaning	Variable definition
Dependent variables	Depression index 1	cesd20sc (score)
	Depression index 2	cesd8 (score)
Explanatory variable	Internet use	Whether the respondent uses mobile devices (e.g., mobile phone, tablet) to access the internet: 1 = yes; 0 = no;
		Whether the respondent uses the internet: 1 = yes; 0 = no
Instrumental variable	Internet access of other residents within the region	The internet access rate of other villagers or community residents (number of people using the internet ÷ total number of people)
Mediating variable	Exercise frequency	Participation frequency in physical exercise or recreational sports activities during the past 12 months:
		0. Never
		1. On average, less than once per month
		2. On average, at least once per month but less than once per week
		3. On average, 1–2 times per week
		4. On average, 3–4 times per week
		5. On average, 5 times per week or more
		6. Once per day
		7. Twice per day or more
Control variable	Gender	1 = male; 0 = female
	Age in years	Age at the time of survey (years)
	Registered residence type	1 = rural; 0 = urban
	Education	Years of education
	Marital status	1 = married; 0 = unmarried
	Employment status	Whether the respondent has a full-time job: 1 = yes; 0 = no
	Household income	Total household income (log-transformed)
	Household size	Number of household members
	Health status	Self-rated health status
		1 = BMI within normal range (18.5 ≤ BMI ≤ 23.9); 0 = BMI outside normal range (BMI < 18.5 or BMI ≥ 23.9)

Descriptive statistics for the variables are presented in Table 2. The sample includes 16,901 observations, and the distributions of the variables appear generally reasonable. For the dependent variables, the mean value of cesd20sc is 33.62 with a standard deviation of 8.241, ranging from 22 to 72. The mean value of cesd8 is 13.77 with a standard deviation of 4.148, ranging from 8 to 32. These results indicate that there is notable variation in depression levels among the respondents.

**Table 2:** Descriptive statistics of variables.

Variables	Variable meaning	Mean	Std. Dev.	Min	Max
Dependent variables	Depression index 1	33.62	8.241	22	72
	Depression index 2	13.77	4.148	8	32
Explanatory variable	Mobile internet use	0.720	0.449	0	1
	Internet use	0.723	0.448	0	1
Instrumental variable	Internet access of other residents within the region	0.723	0.166	0	1
Mediating variable	Exercise frequency	1.637	2.326	0	7
Control variables	Gender	0.550	0.498	0	1
	Age in years	46.66	12.94	18	70
	Registered residence type	0.831	0.375	0	1
	Years of education	9.444	4.516	0	22
	Marital status	0.828	0.377	0	1
	Employment status	0.811	0.391	0	1
	Household income	11.14	1.156	0	16.59
	Household size	3.659	1.900	1	15
	Self-rated health status	3.049	1.154	1	5
	BMI in normal range	0.519	0.500	0	1

For the explanatory variables, the mean values of the two indicators measuring internet use are 0.720 and 0.723, indicating that approximately 72% of the respondents use the internet or access it via mobile devices, while about 28% do not use the internet at all. The mediating variable, exercise frequency, has a mean of 1.639 and a standard deviation of 2.319, suggesting substantial variation in residents’ exercise habits and indicating that most respondents do not engage in physical activity frequently.

In terms of control variables, the mean value of gender is 0.548, indicating a slightly higher proportion of men than women. The mean age is 46.66 years, indicating that the sample consists primarily of adults and middle-aged or older individuals. The mean value of household registration type is 0.830, showing a higher proportion of rural residents. The average years of education is 9.444, which approximates the level of junior secondary schooling. The mean value of marital status is 0.822, suggesting that married residents constitute the majority. The mean value of employment status is 0.813, indicating that most residents hold full-time jobs. The mean of the logarithm of household income is 11.15, and the average household size is 3.659 persons. Regarding health indicators, the mean value of self-rated health status is 3.061 on a five-point scale, the proportion of normal BMI is 0.521, and the distribution of health status across the sample is relatively balanced.

### 4.2 Econometric models

To examine the impact of mobile internet use on mental health and explore the pathway linking mobile internet use, physical exercise frequency, and depressive symptoms, this study first estimates a two-way fixed-effects model and then examines the mediating role of exercise frequency.

First, Model (1) is constructed to examine the direct impact of mobile internet use on residents’ mental health:


$$C{\mathrm{esd}}_{\mathrm{it}}=\alpha +\alpha_{1}M\_{\mathrm{web}}_{\mathrm{it}}+\alpha_{2}X_{\mathrm{it}}+\delta_{i}+\theta_{t}+\varepsilon_{\mathrm{it}}$$ (1)

Second, taking the frequency of physical exercise as the dependent variable, Model (2) is constructed to examine the effect of mobile internet use on residents’ exercise frequency:


$$E\_{\text{freq}}_{\mathrm{it}}=\beta +\beta_{1}M\_{\mathrm{web}}_{\mathrm{it}}+\beta_{2}X_{\mathrm{it}}+\delta_{i}+\theta_{t}+\varepsilon_{\mathrm{it}}$$ (2)

Finally, Model (3) includes both mobile internet use and physical exercise frequency to assess whether exercise frequency may serve as a potential mediating pathway linking mobile internet use to residents’ mental health:


$$C{\mathrm{esd}}_{\mathrm{it}}=\gamma +\gamma_{1}M\_{\mathrm{web}}_{\mathrm{it}}+\gamma_{2}E\_{\text{freq}}_{\mathrm{it}}+\gamma_{3}X_{\mathrm{it}}+\delta_{i}+\theta_{t}+\varepsilon_{\mathrm{it}}$$ (3)

where *C*esd*_it_* denotes the depression scale score of individual *i* in year *t*; *M_*web*_it_* indicates whether individual *i* used mobile internet in year *t*; *E_*freq*_it_* represents the exercise frequency of individual *i* in year *t*; and *X_it_* is a set of control variables, including gender, age, years of education, marital status, household registration type, employment status, household size, household income, and health status. *δ_i_* denotes the region fixed effects, *θ_t_* denotes the time fixed effects, and *ε_it_* is the error term.

### 4.3 Discussion of endogeneity

To address potential reverse causality between mobile internet use and depressive symptoms, as well as possible omitted-variable bias, this study uses the internet access rate of other villagers or community residents as an instrumental variable reflecting the overall level of internet penetration in the community. The rationale for selecting this variable as an instrument is based on two considerations: first, regarding relevance, the Internet access status of other residents in the same community can influence an individual’s mobile internet use through social network effects, information diffusion, and similar mechanisms, thereby meeting the requirement that the instrumental variable must be correlated with the core explanatory variable; second, concerning exogeneity, an individual’s depressive symptoms constitute a micro-level psychological state that does not exert a reverse influence on the community-level Internet penetration rate, and this variable is unlikely to be affected by other unobserved individual characteristics, thus satisfying the assumption that the instrumental variable is exogenous to the error term. This approach helps mitigate endogeneity concerns, improves the credibility of the estimates, and provides a stronger basis for interpreting the impact of mobile internet use on depressive symptoms.

## 5 Results

### 5.1 The impact of mobile internet use

Table 3 reports the baseline regression results of the model. Regardless of whether control variables are included, the estimated coefficients on the core explanatory variable—mobile internet use—are significantly negative at the 1% level, indicating that mobile internet use is associated with lower depressive symptom scores. This result remains unchanged when the dependent variable is alternatively measured using the cesd20sc or cesd8 score. Without control variables (Columns 1 and 3), the regression coefficient of mobile internet use is −1.389 for cesd20sc and −0.694 for cesd8. After including control variables (Columns 2 and 4), the absolute values of the coefficients decrease, with coefficients of −0.654 for cesd20sc and −0.330 for cesd8, both still significant at the 1 percent level, indicating that even after controlling for individual and household characteristics, the depression-reducing effect of internet use remains robust.

**Table 3:** Benchmark regression results.

Variables	(1)	(2)	(3)	(4)
	cesd20sc score	cesd20sc score	cesd8 score	cesd8 score
Mobile internet use	−1.389^***^ (0.174)	−0.654^***^ (0.181)	−0.694^***^ (0.087)	−0.330^***^ (0.091)
Gender		−1.009^***^ (0.145)		−0.509^***^ (0.073)
Age		−0.067^***^ (0.007)		−0.034^***^ (0.004)
Years of education		−0.232^***^ (0.020)		−0.116^***^ (0.010)
Health status		−2.088^***^ (0.063)		−1.052^***^ (0.032)
Marital status		−2.571^***^ (0.225)		−1.291^***^ (0.113)
Employment status		0.522^**^ (0.184)		0.269^**^ (0.092)
Registered residence type		0.140 (0.190)		0.076 (0.096)
Household size		−0.027 (0.043)		−0.014 (0.022)
Household income (log)		−0.391*** (0.071)		−0.197*** (0.036)
Constant	34.620*** (0.153)	52.391*** (0.904)	14.272*** (0.077)	23.228*** (0.454)
Number of observations	16,142	16,142	16,142	16,142
*R* ^2^	0.090	0.207	0.090	0.207
Adj-*R*^2^	0.041	0.164	0.042	0.164
Year FE	Yes	Yes	Yes	Yes
County FE	Yes	Yes	Yes	Yes

*, **, *** denote significance at the 0.1, 0.05, and 0.01 level, respectively.

The estimated effects of the control variables are generally consistent with theoretical expectations. Male residents exhibit significantly lower levels of depression than females, which may be related to gender differences in emotional expression and social role allocation. Age is significantly negatively associated with depressive symptoms, meaning that depression tends to decrease slightly as individuals grow older, possibly because accumulated life experience facilitates psychological adjustment. Years of education and logged household income both have significant negative associations with depressive symptoms; higher educational attainment and income may alleviate depressive symptoms by enhancing cognitive resources and reducing financial stress. Better self-rated health and being married are associated with significantly lower levels of depression, as good physical condition and stable marital relationships provide psychological support and help mitigate depressive feelings. Residents with full-time employment exhibit significantly higher depression levels than those without full-time jobs, which may be related to work pressure and workplace competition. Urban–rural differences and household size do not appear to be major determinants of depressive symptoms in this sample.

### 5.2 IV regression results

Table 4 reports the first-stage and second-stage regression results of the instrumental variable estimation for the effect of internet use on the cesd20sc score. The first-stage regressions, shown in Columns (1a) and (2a), indicate that the instrumental variable—the internet access rate of other villagers or community residents—is significantly associated with the core explanatory variable, indicating a strong correlation between the instrumental variable and internet use. The Kleibergen–Paap rk Wald F statistics are 194.961 and 169.892, both far exceeding the critical value of 16.38 under a 10 percent maximal relative bias, and the *p*-values of the Kleibergen–Paap rk LM test are both 0.0000, suggesting that the instrumental variable satisfies the relevance and identification assumptions, with no problems of under-identification or weak instruments; thus, the chosen instrument is valid. The second-stage regressions in Columns (1b) and (2b) show that mobile internet use significantly reduces the cesd20sc score. Whether control variables are included or not, the estimated coefficients of the core explanatory variable—mobile internet use—remain significantly negative, indicating that after addressing endogeneity and controlling for individual and household characteristics, the depression-reducing effect of internet use remains robust.

**Table 4:** Instrumental variable regression results.

Variables	(1a) mobile internet use	(1b) cesd20sc score	(2a) mobile internet use	(2b) cesd20sc score
Other villagers’ (community residents’) internet access	−4.268*** (0.306)		−3.098*** (0.238)	
Mobile internet use		−1.783*** (0.357)		−1.349** (0.479)
Gender			−0.001 (0.006)	−1.011*** (0.145)
Age			−0.012*** (0.000)	−0.077*** (0.009)
Years of education			0.020*** (0.001)	−0.217*** (0.023)
Health status			−0.004 (0.003)	−2.091*** (0.063)
Marital status			0.062*** (0.009)	−2.526*** (0.227)
Employment status			−0.031*** (0.008)	0.505** (0.184)
Registered residence type			−0.046*** (0.008)	0.106 (0.191)
Household size			−0.006** (0.002)	−0.032 (0.043)
Household income (log)			0.037*** (0.004)	−0.363*** (0.073)
Number of observations	16,142	16,142	16,142	16,142
*R* ^2^	0.254	0.005	0.437	0.133
Adj-*R*^2^	0.214	−0.048	0.407	0.086
Year FE	Yes	Yes	Yes	Yes
County FE	Yes	Yes	Yes	Yes
Kleibergen–Paap rk Wald F	194.961 [16.38]		169.892 [16.38]	
Kleibergen–Paap rk LM	*p* = 0.0000		*p* = 0.0000	

*, **, *** denote significance at the 0.1, 0.05, and 0.01 level, respectively.

### 5.3 Heterogeneity analysis

To examine group heterogeneity in the effect of internet use on depressive symptoms, this study conducts a heterogeneity analysis from three dimensions—BMI status, employment type, and pension coverage—and the regression results are presented in Table 5.

**Table 5:** Heterogeneity analysis.

Variables	(1)	(2)	(3)	(4)	(5)	(6)	(7)	(8)
	cesd20sc score							
	Bmigood group	Bmibad group	Agricultural Work	Non-agricultural Work	Knowledge-intensive industry	Non-knowledge-intensive industry	With Pension	Without Pension
Mobile internet use	−0.440 (0.256)	−0.867*** (0.256)	0.099 (0.285)	−1.136*** (0.270)	−0.139 (0.802)	−0.654*** (0.188)	−0.397 (0.244)	−0.848** (0.284)
Gender	−0.972*** (0.202)	−0.980*** (0.214)	−0.573** (0.184)	−1.734*** (0.270)	−0.243 (0.381)	−1.204*** (0.160)	−1.100*** (0.184)	−0.907*** (0.237)
Age	−0.065*** (0.010)	−0.069*** (0.010)	−0.080*** (0.010)	−0.051*** (0.014)	−0.130*** (0.021)	−0.063*** (0.008)	−0.041*** (0.011)	−0.078*** (0.010)
Years of education	−0.251*** (0.029)	−0.210*** (0.029)	−0.248*** (0.028)	−0.193*** (0.036)	−0.311*** (0.066)	−0.211*** (0.023)	−0.201*** (0.027)	−0.261*** (0.032)
Health status	−2.165*** (0.090)	−2.018*** (0.091)	−2.047*** (0.088)	−2.008*** (0.103)	−2.055*** (0.201)	−2.083*** (0.067)	−1.995*** (0.081)	−2.167*** (0.101)
Marital status	−2.164*** (0.292)	−2.996*** (0.353)	−2.263*** (0.282)	−3.806*** (0.479)	−1.538** (0.537)	−2.724*** (0.251)	−2.942*** (0.302)	−2.302*** (0.342)
Employment status	0.365 (0.255)	0.604* (0.268)	0.008 (0.273)	0.285 (0.460)	0.150 (0.593)	0.563** (0.199)	0.009 (0.296)	0.742** (0.259)
Registered residence type	0.243 (0.264)	0.214 (0.276)	−0.059 (0.226)	0.229 (0.636)	−0.505 (0.417)	0.236 (0.220)	0.233 (0.238)	0.019 (0.317)
Household size	−0.102 (0.059)	0.048 (0.064)	−0.011 (0.060)	0.013 (0.072)	0.088 (0.121)	−0.041 (0.046)	−0.012 (0.059)	−0.039 (0.063)
Household income (log)	−0.308** (0.096)	−0.464*** (0.110)	−0.436*** (0.108)	−0.412*** (0.121)	−0.413 (0.236)	−0.403*** (0.075)	−0.342*** (0.098)	−0.417*** (0.105)
Constant	51.863*** (1.259)	52.702*** (1.355)	52.787*** (1.333)	53.321*** (1.754)	54.395*** (2.947)	52.360*** (0.970)	50.643*** (1.227)	53.452*** (1.355)
Number of observations	8,233	7,600	9,239	5,238	2,214	13,675	9,500	6,351
*R* ^2^	0.217	0.231	0.208	0.229	0.295	0.213	0.217	0.240
Adj-*R*^2^	0.151	0.170	0.136	0.185	0.114	0.169	0.156	0.174
Year FE	Yes	Yes	Yes	Yes	Yes	Yes	Yes	Yes
County FE	Yes	Yes	Yes	Yes	Yes	Yes	Yes	Yes

*, **, *** denote significance at the 0.1, 0.05, and 0.01 level, respectively.

Among these groups, mobile internet use has a significant depression-reducing effect on residents with abnormal BMI (including overweight, obesity, or underweight). On the one hand, digital technology provides them with more convenient channels for social interaction, helping to break social isolation and rebuild emotional connections, thereby alleviating the sense of social exclusion that may arise from body-shape concerns. On the other hand, the Internet enables access to extensive professional information about body management and exercise, which lowers the barriers to action and provides clearer guidance for behavioral adjustments. Such clarity can substantially reduce depressive symptoms stemming from self-doubt, thus exerting a significant negative impact on depression levels.

Second, the depression-reducing effect of mobile internet use is stronger among non-agricultural workers. This may stem from the close alignment between internet functions and the high-pressure, fast-paced nature of their work. The Internet’s informational and entertainment functions can meet users’ needs for quick access to information and entertainment in their spare moments, alleviate work-related irritability, anxiety, and other negative emotions in a timely manner, and expand channels for exercise, social interaction, and emotional expression, all of which play a positive role in improving emotional states and reducing depressive risk. In contrast, although agricultural work is physically demanding, its rhythm is often synchronized with natural cycles and tends to be more regular and autonomous. Moreover, farmers’ social support networks may rely more on real-life interactions with neighbors, relatives, and local communities rather than virtual online networks, resulting in a nonsignificant effect on their depression levels.

To further probe the role of occupational type, we refine the occupational classification by distinguishing between knowledge-intensive industries, including information technology, finance, education, healthcare, scientific research, and public administration, and non-knowledge-intensive industries comprising manufacturing, construction, wholesale and retail, and related sectors. This classification is grounded in the National Bureau of Statistics’ Classification of Knowledge-Intensive Service Industries (2020) and OECD standards, and reflects fundamental differences in knowledge density, work mode, and patterns of internet engagement across occupations.

The results show that mobile internet use exerts a significant depression-reducing effect among workers in non-knowledge-intensive industries, whereas the effect is statistically non-significant among those in knowledge-intensive industries. Workers in knowledge-intensive industries tend to engage in high-frequency, work-obligatory internet use for much of their workday. Such obligatory use may generate cognitive fatigue and digital overload rather than psychological relief, thereby attenuating or even offsetting the mental health benefits that might otherwise arise from discretionary internet use. In contrast, workers in non-knowledge-intensive industries are less likely to be saturated by work-related internet demands, and their mobile internet use is therefore more likely to take the form of voluntary, leisure-oriented, or health-seeking activity, precisely the mode of use most conducive to promoting physical exercise and alleviating depressive symptoms. This finding provides indirect empirical support for the theoretical distinction between active and passive internet use. Taken together, the occupational heterogeneity results suggest that the mental health benefits of mobile internet use are contingent not merely on access, but on the degree to which individuals can engage with digital technologies in discretionary, health-oriented ways.

Notably, the depression-alleviating effect of internet use is more pronounced among residents without pension coverage. Compared with individuals who possess pension security, this group often faces greater uncertainty and emotional stress when contemplating their future livelihood. In this context, Internet platforms play an important role in psychological adjustment. On the one hand, through government service platforms, short videos, public accounts, and similar channels, the Internet lowers the informational threshold for understanding pension-related policies, enabling individuals without pension coverage to obtain clearer and timelier policy information, thereby easing confusion and anxiety caused by information asymmetry. On the other hand, the Internet offers online social spaces and virtual support networks that help individuals rebuild a sense of social connectedness through emotional support and experience sharing, partially compensating for the lack of offline social support systems. These mechanisms jointly form a psychological buffering channel for groups without pension coverage, thereby strengthening the regulatory effect of internet use on their depressive symptoms. In contrast, when individuals’ basic pension needs are already secured by institutional arrangements, the marginal alleviating effect of internet use becomes relatively weaker.

### 5.4 Exploratory mediation analysis

To explore the potential behavioral pathway through which mobile internet use may be associated with residents’ depressive symptoms, this study selects exercise frequency as a mediating variable and conducts an exploratory mediation analysis using the causal steps approach. It should be emphasized that the mediation analysis in this section is exploratory in nature and is intended to identify a plausible transmission mechanism rather than to establish a definitive causal mediation effect. The regression results are presented in Table 6.

**Table 6:** Exploratory mediation regression results.

Variables	(1)	(2)	(3)	(4)
	Exercise frequency	cesd20sc score	Exercise frequency	cesd20sc score
Mobile internet use	0.369^***^ (0.046)	−1.276^***^ (0.174)	0.449^***^ (0.051)	−0.569^**^ (0.181)
Exercise frequency		−0.306^***^ (0.031)		−0.189^***^ (0.030)
Gender			0.098^*^ (0.040)	−0.991^***^ (0.144)
Age			0.040^***^ (0.002)	−0.060^***^ (0.007)
Years of education			0.096^***^ (0.006)	−0.214^***^ (0.021)
Health status			0.070^***^ (0.017)	−2.075^***^ (0.063)
Marital status			−0.190^**^ (0.058)	−2.607^***^ (0.225)
Employment status			−0.716^***^ (0.056)	0.387^*^ (0.185)
Registered residence type			−0.416^***^ (0.059)	0.061 (0.190)
Household size			−0.055^***^ (0.012)	−0.037 (0.043)
Household income (log)			0.150^***^ (0.019)	−0.363^***^ (0.071)
Constant	1.371^***^ (0.038)	35.039^***^ (0.159)	−2.089^***^ (0.243)	51.995^***^ (0.907)
Number of observations	16,142	16,142	16,142	16,142
*R* ^2^	0.127	0.096	0.196	0.209
Adj-*R*^2^	0.081	0.048	0.153	0.166
Year FE	Yes	Yes	Yes	Yes
County FE	Yes	Yes	Yes	Yes

*, **, *** denote significance at the 0.1, 0.05, and 0.01 level, respectively.

The preceding analysis has shown that mobile internet use is significantly negatively associated with depressive symptom scores, providing a preliminary basis for exploring possible mediating pathways. Columns 1 and 3 of Table 6 report the association between mobile internet use and exercise frequency. The results show that, regardless of whether control variables are included, mobile internet use is significantly positively associated with exercise frequency. This finding suggests that residents who use the mobile Internet tend to engage in physical exercise more frequently.

Columns 2 and 4 of Table 6 further include both mobile internet use and exercise frequency in the regression models of depressive symptom scores. The results indicate that mobile internet use remains significantly negatively associated with depressive symptoms after exercise frequency is added to the model. Meanwhile, exercise frequency is also significantly negatively associated with depressive symptom scores. In the specification with control variables, the coefficient of mobile internet use decreases in magnitude after the mediating variable is included, compared with the corresponding baseline model without exercise frequency. This pattern suggests that exercise frequency may partially account for the relationship between mobile internet use and residents’ depressive symptoms. Therefore, the results provide exploratory evidence that exercise frequency may serve as a partial mediating pathway linking mobile internet use to lower depressive symptom levels.

### 5.5 Robustness test

To verify the reliability of the baseline regression results, this study conducts a robustness check by replacing the explanatory variable (Table 7). The original indicator of “mobile internet use” is replaced with the indicator of “internet use,” and the regression results show that the core conclusions remain valid. In Column (1), the regression coefficient of the substituted internet use variable on the depression score cesd20sc is −0.691 and significantly negative at the 1 percent level, indicating a clear depression-reducing effect of internet use. Column (2) shows that the regression coefficient of the substituted internet use variable on the mediating variable (exercise frequency) is 0.460 and significantly positive at the 1 percent level, confirming the robustness of the Internet’s promoting effect on exercise frequency. Column (3), which simultaneously includes the substituted internet use variable and exercise frequency, shows that the regression coefficient of internet use on cesd20sc is −0.604 (significant at the 1 percent level), with an absolute value smaller than −0.691 in Column (1), and the regression coefficient of exercise frequency is −0.189 (significant at the 1 percent level). These findings again provide supportive evidence consistent with a partial mediating role of exercise frequency. The directions and significance levels of the control variables remain unchanged, providing further evidence that the core conclusions of this study are robust.

**Table 7:** Robustness check results (alternative explanatory variable).

Variables	(1)	(2)	(3)
	cesd20sc score	Exercise frequency	cesd20sc score
Internet use	−0.691*** (0.182)	0.460*** (0.051)	−0.604*** (0.183)
Exercise frequency			−0.189*** (0.030)
Gender	−1.009*** (0.145)	0.097* (0.040)	−0.991*** (0.144)
Age	−0.068*** (0.007)	0.040*** (0.002)	−0.060*** (0.007)
Years of education	−0.231*** (0.020)	0.095*** (0.006)	−0.213*** (0.021)
Health status	−2.088*** (0.063)	0.070*** (0.017)	−2.075*** (0.063)
Marital status	−2.569*** (0.225)	−0.191** (0.058)	−2.605*** (0.225)
Employment status	0.521** (0.184)	−0.716*** (0.056)	0.386* (0.185)
Registered residence type	0.137 (0.190)	−0.415*** (0.059)	0.059 (0.190)
Household size	−0.027 (0.043)	−0.055*** (0.012)	−0.038 (0.043)
Household income (log)	−0.389*** (0.071)	0.149*** (0.019)	−0.361*** (0.071)
Constant	52.415*** (0.904)	−2.095*** (0.243)	52.019*** (0.907)
Number of observations	16,142	16,142	16,142
*R* ^2^	0.207	0.196	0.209
Adj-*R*^2^	0.164	0.153	0.166
Year FE	Yes	Yes	Yes
County FE	Yes	Yes	Yes

*, **, *** denote significance at the 0.1, 0.05, and 0.01 level, respectively.

Table 8 presents the robustness test results obtained by conducting 1,000 Bootstrap replications to further verify the validity of the mediating mechanism of exercise frequency. The results show that the 95% confidence interval for the indirect effect does not include zero, suggesting that the association between mobile internet use and depressive symptoms may partly operate through increased exercise frequency. This finding is consistent with the preceding exploratory mediation analysis and provides further suggestive evidence that exercise frequency may serve as a partial behavioral pathway linking mobile internet use to residents’ depressive symptoms.

**Table 8:** Bootstrap estimation results of indirect effects.

Statistic	Estimate	*z*-value	*p* > |*z*|	95% confidence interval
Direct effect	−0.391	−2.31	0.021	[−0.723, −0.059]
Indirect effect	−0.092	−5.75	0.000	[−0.124, −0.061]

Based on the assumption that the relationship between the treatment effect and unobservable variables can be inferred from its relationship with observable variables, Oster (58) argues that the absolute value of the estimated coefficient of the core explanatory variable typically decreases after adding more control variables, implying that even if unobservable omitted variables exist, their influence on the estimation results is likely limited. Although this study has addressed endogeneity concerns and incorporated two-way fixed effects, it cannot completely eliminate the endogeneity arising from unobservable omitted variables. Therefore, to further mitigate the potential influence of such factors, this study applies the coefficient stability test proposed by Oster (58), and the test results are presented in Table 9. The specific procedures are as follows: first, *R*_max_ is set to 1.3 times the baseline regression *R*^2^ (0.269), and *δ* is set to 1, after which the feasible range of the adjusted coefficient *β*^∗^ for the core explanatory variable is estimated; second, with *R*_max_ set to 1.3 times the baseline regression *R*^2^ (0.269), *β^∗^* is set to 0, and the value of *δ* is calculated accordingly. The first row of Table 9 reports the results of the first procedure, where the range of *β*^∗^ does not include zero, indicating that the test is passed. The second row corresponds to the second procedure, and if the calculated *δ* exceeds 1, the test is considered passed. The results in Table 9 show that both procedures successfully pass the test, thereby confirming the robustness of the main conclusions of this study—namely, that omitted variables do not affect the validity of the empirical results.

**Table 9:** Coefficient stability test results.

Method	Parameter setting	Judgment criterion	Actual estimation result	Passed or no
(1)	*R*_max_ = 0.269, *δ* = 1	The interval does not contain 0	[−1.603, −0.691]	Yes
(2)	*R*_max_ = 0.269, *β*^∗^ = 0	The value of δ is greater than 1	1.460	Yes

## 6 Discussion

Against the backdrop of the continuously increasing global mental health burden and the accelerating pace of digitalization, how to improve residents’ mental health with the aid of digital technologies has become an important issue in the fields of public health and social governance. Using data from the CFPS for 2020–2022, this paper adopts a behavioral mediation perspective to systematically examine the effect of mobile internet use on residents’ depression levels, with a particular focus on the transmission role played by the frequency of physical exercise. By employing a two-way fixed effects model, an instrumental variable approach, and multiple robustness checks, this paper substantially addresses potential endogeneity concerns, thereby providing more robust empirical evidence for understanding the mechanism of “mobile internet–behavioral change–mental health.” This section further discusses the implications of the findings and situates them within the broader literature.

First, we re-examine the relationship between mobile internet use and depression levels. The empirical results show that mobile internet use significantly reduces residents’ depression levels; this conclusion remains robust after controlling for individual characteristics, household characteristics, regional fixed effects, and time fixed effects, and is further confirmed after using an instrumental variable method to address potential reverse causality and omitted variable bias. This finding provides strong support for the research strand arguing that “internet use has mental health–promoting effects” (15, 20, 59), and to some extent responds to the debate in the existing literature regarding whether internet use may exacerbate depression (23, 24). Unlike studies that emphasize the risks of “problematic internet use” (22), our results indicate that, at the aggregate level, mobile internet use is more of a psychological resource than a psychological burden. This may be because mobile internet use has become deeply embedded in residents’ daily behavioral structures in real life, and its core functions include not only information acquisition and entertainment, but, more importantly, the provision of emotional support, social connectedness, and health management tools (16, 21). Especially against the backdrop of an accelerated social pace and generally rising psychological stress, mobile internet use lowers the barriers to accessing psychological support and health information, thereby serving as an emotional buffer (53, 60, 61).

Second, the exploratory mediation results suggest that mobile internet use may be related to depressive symptoms not only directly but also indirectly through increased physical exercise frequency. One possible explanation is that mobile internet use provides residents with access to health-related information, exercise guidance, digital fitness resources, and opportunities for online social interaction, all of which may facilitate the initiation and maintenance of physical activity (35, 36). This finding is also consistent with Behavioral Activation Theory (43). Self-Determination Theory offers a possible psychological explanation for this mechanism. Through personalized recommendations and data feedback, mobile internet use enhances individuals’ sense of competence; by allowing flexible choices of exercise modes, it satisfies autonomy; and through online communities and social platforms, it strengthens relatedness, thereby increasing intrinsic motivation for physical exercise and sustaining behavioral persistence (47). As a result, physical exercise becomes a form of psychological regulation that may contribute to emotional improvement (62). However, given the exploratory nature of the mediation analysis, this pathway should be interpreted with caution. Future experimental or quasi-experimental studies are needed to establish the causal status of physical exercise as a mediating mechanism.

The theoretical justification for selecting physical exercise as the focal mediating variable, rather than other plausible alternatives, warrants brief elaboration here. Among the behavioral pathways through which mobile internet use may influence mental health, physical exercise is uniquely supported by all three theoretical frameworks underpinning this study. Physical exercise constitutes a prototypical health-enhancing behavior aligned with Uses and Gratifications Theory by satisfying users’ informational and social needs through digital resources, a positive rewarding activity consistent with Behavioral Activation Theory by interrupting the behavioral inhibition-negative affect cycle of depression, and a domain where mobile internet fulfils the three basic psychological needs specified by Self-Determination Theory. Nevertheless, physical exercise represents only one of several potential behavioral transmission channels. Other pathways may operate in parallel and deserve systematic investigation in future research.

Third, this paper also discusses group heterogeneity and digital health inequalities. The heterogeneity analysis reveals structural differences in the mental health effects of mobile internet use. For individuals with abnormal BMI, pressures related to body image and health anxiety tend to be more salient. Mobile internet use, by providing body management knowledge, exercise programs, and reference points for social comparison, helps reduce self-denial and emotional distress, thereby more effectively alleviating depressive symptoms (63, 64). For non-agricultural workers, who typically face faster work rhythms and higher levels of stress, mobile internet use has a greater marginal effect in relieving fragmented time pressure and expanding channels for emotional regulation. The refined occupational analysis further corroborates this interpretation. Among workers in non-knowledge-intensive industries, mobile internet use exerts a significant depression-reducing effect, consistent with the pattern observed for non-agricultural workers more broadly. For individuals lacking pension security, psychological anxiety stemming from future uncertainty is particularly pronounced, and the Internet’s functions in information acquisition, policy understanding, and social support make it an important psychological “buffer” (65–67). These findings indirectly confirm that digital health promotion is not merely a technological issue, but is deeply embedded in social structures, reflecting pronounced effects of social stratification (68).

A potential concern warrants clarification here. Given that pension coverage rates tend to be lower among agricultural workers, do the effects on these two groups present a contradiction? We argue that these two findings are not contradictory because they reflect fundamentally different mechanisms operating at different analytical levels. The pension coverage dimension reflects psychological uncertainty about future economic security: individuals without pension protection face heightened anxiety about livelihood, and mobile internet use alleviates this by improving access to policy information and virtual social support networks. The occupational dimension, by contrast, reflects differences in work rhythm, stress structure, and the mode of internet engagement: agricultural workers’ depression is less responsive to internet use not because they lack economic anxiety, but because their social support needs are predominantly met through offline community ties, and their pattern of internet use is less likely to take forms conducive to mental health promotion. These two dimensions are therefore conceptually orthogonal, and their apparently divergent findings reflect the multidimensional nature of social vulnerability.

Finally, from a methodological standpoint, this study advances beyond much of the existing literature in three respects. First, unlike studies relying on cross-sectional data (20, 38, 50–52), the use of panel data allows for the control of time-invariant individual heterogeneity, providing a more credible basis for causal inference. Second, the application of instrumental variable estimation addresses the reverse causality concern that individuals with lower depression may be more inclined to use mobile internet, a source of bias that most prior studies have not systematically resolved (19, 53). Third, rather than treating mobile internet use as exerting a purely direct effect (37, 50–53), this study conducts an exploratory mediation analysis to examine whether its association with psychological well-being may operate partly through a behavioral pathway. By identifying physical exercise frequency as a plausible, but not definitive, mediating mechanism, the study provides a more nuanced account of how digital engagement may be linked to residents’ mental health outcomes.

## 7 Limitations

Despite its contributions, this study has several limitations that should be acknowledged.

First, mobile internet use is measured using a binary indicator that distinguishes only between users and non-users. This approach cannot capture variation in usage intensity, frequency, or purpose, and in particular cannot distinguish between active, voluntary engagement and passive or work-obligatory use. The occupational heterogeneity results provide indirect evidence that this distinction matters, but direct empirical testing requires more granular data such as purpose-specific usage scales or digital trace records. Nevertheless, the binary indicator remains a feasible and informative measure, enabling identification of the core association between mobile internet access and depressive symptoms, a reasonable trade-off given the constraints of available data. Future research could draw on more finely disaggregated data to develop more refined measures that better account for internal heterogeneity in mobile internet use.

Second, the mediation analysis follows a causal steps approach supplemented by Bootstrap estimation. While the results are consistent with a partial mediating role of physical exercise, causal interpretation of indirect effects remains inherently limited in observational settings. Causal inference should still be interpreted with caution. Future research could adopt randomized experiments or more rigorous instrumental variable strategies for further validation.

Third, this study examines physical exercise as a single behavioral transmission channel. This choice is theoretically motivated and empirically feasible given the available data, but other potential mediating pathways—including social participation, sleep quality, and dietary behavior—may operate in parallel and deserve systematic investigation in future research.

In addition, the study period from 2020 to 2022 overlaps with the COVID-19 pandemic. The core estimates should therefore be interpreted with this contextual caveat in mind. Concerning the confounding effects of the pandemic period, although individual-level pandemic-related variables are unavailable, China’s highly unified national epidemic prevention and control framework implies that core policies were implemented synchronously across regions. Year fixed effects capture the time-varying impacts of nationwide policies, while county fixed effects account for minor regional differences in policy implementation; therefore, unobserved individual-level pandemic factors have limited interference with the core causal relationship examined in this study.

Overall, the existing literature has limitations in terms of variable selection and methodological design. Constrained by available data, this study has minimized potential bias to the greatest extent possible through its research design, and further improvements can be made in future research.

## 8 Conclusion and implications

This study examines the relationship between mobile internet use and resident’ depressive symptoms from the perspective of behavioral mechanisms. By incorporating the frequency of physical exercise into the mediating pathway and employing a two-way fixed effects model with instrumental variables to address endogeneity concerns, the findings provide robust empirical evidence that mobile internet use contributes to improvements in individuals’ mental health. The exploratory mediation analysis further suggests that mobile internet use may be linked to reduced depressive symptoms by reshaping individuals’ daily behavioral environments and promoting participation in health-enhancing behaviors. Nevertheless, because the mediation analysis is exploratory, this pathway should be interpreted with caution and should not be taken as conclusive evidence of a causal mediation effect.

From a broader perspective, the findings reveal the socially embedded and heterogeneous nature of the digital health effect. The mental health benefits associated with mobile internet use are more pronounced among socially or economically vulnerable groups, suggesting that digital technologies may function as compensatory resources under conditions of structural disadvantage. These findings underscore the necessity of moving beyond a technologically deterministic understanding of internet use and adopting more nuanced and contextualized analytical frameworks to elucidate how digital access interacts with behavioral practices and social contexts to jointly shape individuals’ psychological well-being.

These findings carry significant policy implications across several dimensions. For individuals with abnormal BMI, public health authorities and digital platform operators should co-develop integrated body management and mental health tools—such as personalised exercise programmes and peer support communities embedded within mobile applications—that simultaneously address physical health anxiety and depressive risk. For non-agricultural and non-knowledge-intensive workers, employers and occupational health agencies should promote workplace-based digital wellness interventions, including mobile-accessible fitness resources and flexible exercise scheduling. For knowledge-intensive workers whose depression shows weaker responsiveness to mobile internet use, policy should focus on encouraging deliberate digital detachment outside working hours and facilitating access to offline physical activity, recognising that work-saturated internet engagement does not automatically confer mental health benefits. For residents lacking pension coverage, government platforms should prioritise the accessibility of pension-related policy information, while community-level programmes should build digital literacy skills to help this group utilise online social support networks effectively.

## Data Availability

The original contributions presented in the study are included in the article/supplementary material, further inquiries can be directed to the corresponding author.

## Footnotes

1 Given that the study period (2020–2022) covers the COVID-19 pandemic, regional lockdown policies, travel restrictions, and public health events may have affected residents’ internet use, exercise behavior, and mental health. The inclusion of county fixed effects controls for the heterogeneous impacts of region-specific pandemic prevention policies, while year fixed effects control for the time-varying impacts of different pandemic stages.
